# Cushing's Disease Associated With Partially Empty Sella Turcica Syndrome: A Case Report

**DOI:** 10.7759/cureus.40115

**Published:** 2023-06-08

**Authors:** Tehmina Habib, Mohammad Abu-Abaa, Navid Redjal

**Affiliations:** 1 Internal Medicine, Capital Health Regional Medical Center, Trenton, USA; 2 Neurosurgery, Capital Health Regional Medical Center, Trenton, USA

**Keywords:** mri negative cushing disease, empty sella trucica syndrome, pituitary nodule, weight loss, cushing’s disease

## Abstract

The association between empty sella turcica (EST) syndrome and Cushing's disease has been rarely reported. It is plausible to hypothesize that EST syndrome in association with Cushing's disease can be attributed to intracranial hypertension. In this case report, we present a 47-year-old male patient who presented with weight loss, fatigue, easy bruising, acanthosis nigricans, and skin creases hyperpigmentation. Investigations revealed hypokalemia and confirmed the diagnosis of Cushing's disease. Magnetic resonance imaging (MRI) brain showed a partial EST syndrome and a new pituitary nodule as compared with previous brain imaging. Transsphenoidal surgery was pursued and was complicated by cerebrospinal fluid leakage. This case reflects the rare association of EST syndrome and Cushing's disease, suggesting the increased risk of postoperative complications in this setting and the diagnostic challenge that EST syndrome imposes. We review the literature for a possible mechanism of this association.

## Introduction

Cushing's disease is commonly caused by an adrenocorticotropic hormone (ACTH)-producing pituitary adenoma, which can be very challenging to be seen on brain magnetic resonance imaging (MRI) [1]. Empty sella turcica (EST) syndrome is a radiological diagnosis of apparently empty turcica secondary to outpouching of the arachnoid mater into the turcica, which can be attributed to intracranial hypertension (ICHTN). This can make the visual diagnosis of pituitary adenoma even more challenging in clinical practice. ICHTN has been also associated with Cushing's disease and might explain this infrequent association between EST and Cushing's disease [1]. EST syndrome can be either partial or complete, primary or secondary and has been seen infrequently with Cushing's disease. In this setting, not only that it is likely to obscure an underlying pituitary lesion, but also it does contribute to the risk of postoperative complications [2].

## Case presentation

A 47-year-old male presented to the emergency department (ED) with slowly progressive generalized limb muscle weakness affecting both distal and proximal muscles over a few weeks and gait instability for three days prior to presentation. He also reported unintentional 40 pounds weight loss over the previous four months. Past medical history was significant for type II diabetes mellitus, hypothyroidism, hypertension, and dyslipidemia. In the ED, vital signs included a blood pressure of 140/90 mmHg, a heart rate of 66 beats per minute, a respiratory rate of 16 cycles per minute, and SpO_2_ of 97% on room air. Body mass index has decreased to 22 kg/m^2^ from a baseline of 26 kg/m^2^ one month prior. On the physical exam, he exhibited cachexia, easy bruising, acanthosis nigricans, and hyperpigmentation of skin creases. All other systems were negative. Complete metabolic panel and complete blood count were obtained showing hyperglycemia of 311 mg/dl, see Table 1. Further lab evaluation showed elevated salivary cortisol at 2.96 microgram/dl (reference range 0.094-1.551 mcg/dl), elevated 24-hour urinary free cortisol at 156 mcg/24 hour (reference 10-100 mcg/24h), positive overnight dexamethasone suppression test with serum cortisol at 2.8 mcg/dl (reference more than 2 mcg/dl), negative anti-adrenal antibodies, normal aldosterone, and elevated dehydroepiandrostenedione at 401 mcg/dl (reference 32-240 mcg/dl), with lack of suppression of the ACTH level at 35.1 pg/ml (reference 10-60 pg/ml). This confirmed the diagnosis of Cushing's disease.

**Table 1:** Lab Findings

Variable	Finding	Reference
Random glucose	311	Less than 200 mg/dl
Sodium	141	137-145 mmol/L
Potassium	2.5	3.5-5.1 mmol/L
Chloride	96	98-107 mmol/L
Bicarbonate	32	22-30 mmol/L
Blood urea nitrogen	32	9-20 mg/dl
Creatinine	0.52	0.66-1.25 mg/dl
Calcium	8.7	8.6-10.3 mg/dl
Total protein	5.5	6.5-8.5 g/dl
Albumin	3.3	3.5-5 g/dl
Total bilirubin	0.6	0.2-1.3 mg/dl
Alkaline phosphatase	115	38-126 U/L
Aspartate transaminase	17	17-59 U/L
Alanine transaminase	39	Less than 49 U/L
White blood cell count	10x10^3 cells/mcl	4-10x1063 cells/mcl
Hemoglobin	15.3	13.7-17.5 g/dl
Platelet	281	150-400x10^3 cells/mcl

**Figure 1:**
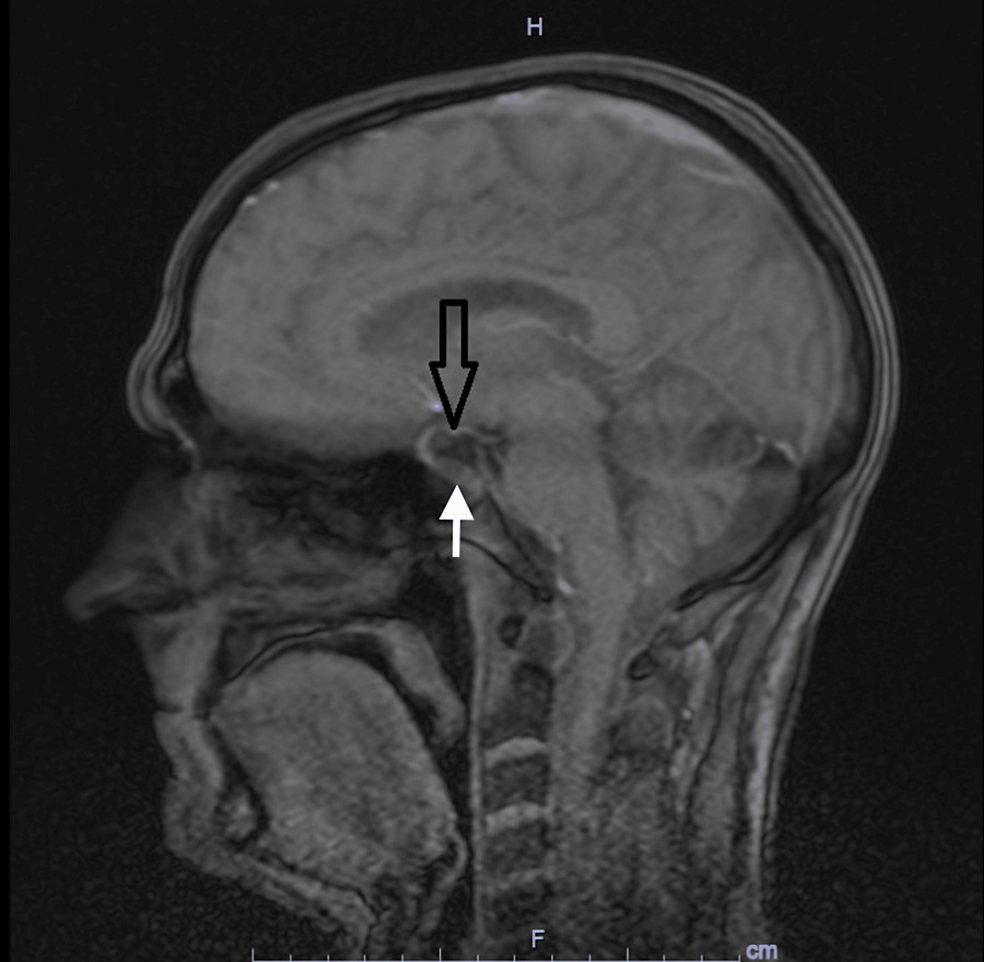
Magnetic Resonance Imaging (MRI) Brain MRI brain showing partially empty sella turcica syndrome ( black arrow) with a small nodule at the floor of the turcica (white arrow).

Computed tomography (CT) scan of the head was unremarkable. CT scan of the chest was also unremarkable. CT scan of abdomen and pelvis showed no adrenal mass. Ultrasound of the kidneys was unremarkable. Pituitary MRI brain protocol for adenoma showed a partial EST, shortening within neurohypophysis and a new 10 x 8 x 4 mm nodule along the floor of pituitary sella as compared to MRI four years ago (Figure 1).

The diagnosis of Cushing’s disease was confirmed, and the patient underwent trans-sphenoidal resection of pituitary adenoma. Histological examination showed positive CAM 5.2, positive chromogranin, and ACTH immunostains. The patient presented to the ED five days after discharge home. He stated that he noticed drainage from the nose that transitioned from bloody to clear fluid and has been increasing in quantity for two days with associated intermittent headaches since the surgery. He was afebrile with stable vital signs. No signs of infection were noted on basic labs. These were significant only for mild asymptomatic hyponatremia of 131 mmol/L and hypokalemia of 3.3 mmol/L. The patient was diagnosed with cerebrospinal fluid (CSF) leakage and had a lumbar drain trial. The trial was unsuccessful after several days, and the patient underwent a transnasal endoscopic repair of CSF rhinorrhea using nasoseptal flaps. At an outpatient follow-up one month and three months after the surgery, prior lab abnormalities including hypokalemia, hyponatremia, and hyperglycemia resolved. No further evidence of CSF leakage was appreciated, and he remained asymptomatic.

## Discussion

EST syndrome is characterized by herniation of the subarachnoid space into the intrasellar space with compression of the pituitary gland into the posteroinferior wall [3]. This is likely to obscure the presence of underlying pituitary mass. The incidence of EST syndrome in the general population is estimated at 20%. The association between EST syndrome and Cushing's disease has been reported infrequently. A retrospective study of 68 patients with Cushing's disease found that 16% of these have EST syndrome [3].

Cushing's disease usually results from pituitary adenomas secreting ACTH, and even the smallest microadenomas can produce a systemic disease. These microadenomas can be very difficult to recognize on brain MRI [4]. This is complicated in EST syndrome and even further with the possibility of ectopic ACTH production. A retrospective study of 197 patients diagnosed with Cushing's disease concluded that EST syndrome is associated with higher prevalence of MRI-negative Cushing's disease. This was attributed to ICHTN and pituitary gland compression [1]. Although surgery is curative in 70-90% of cases, EST syndrome was found to have higher risk of postoperative complications among those with Cushing's disease including diabetes insipidus, hypopituitarism, and CSF leakage [3]. This is usually because in the case of MRI-negative Cushing's disease with total EST syndrome, empiric surgical exploration is sought after inferior petrosal sampling confirms the pituitary origin of excess ACTH, and postoperative remission indicates adequate tumor resection [2]. This entails a higher chance of uncertainty and injury to healthy pituitary tissue. 

EST syndrome can be either primarily due to defects in the sellar diaphragm or anatomical variant or secondary to ICHTN. EST syndrome has been reported in association with many conditions associated with elevated intracranial pressure including tumors, thrombosis, meningitis, hydrocephalus, and Arnold-Chiari malformation [5]. Reversal of EST syndrome has been reported in those with idiopathic ICHTN with therapy by acetazolamide, ventriculoperitoneal shunt, and lumbar puncture [6,7]. A study has shown correlation between CSF circulation impairment or blockage and EST syndrome [8]. The incidence of EST syndrome in association with symptomatic intracranial hypertension is variable and ranges from 2.5% for total EST syndrome to 94% for partial EST syndrome [9]. Impaired CSF circulation and dynamics have been reported in 77% of patients with EST syndrome [10]. In addition to intracranial hypertension, EST syndrome has also been described in association with obesity, meningioma, pediatric nevoid basal cell carcinoma, therapy for growth hormone deficiency and even in healthy individuals [9]. Lack of symptoms of intracranial hypertension in this patient does not rule it out as intracranial hypertension in EST syndrome represents a spectrum that ranges from asymptomatic, milder intracranial hypertension to symptomatic intracranial hypertension with headache, visual disturbance, and papilledema [10]. This explains the fact that only 8-14% of EST syndrome progress to symptomatic ICHTN, while symptomatic ICHTN has been associated with EST syndrome in 94% of cases. 

ICHTN has been seen in association with disturbance of the hypothalamic-pituitary-adrenal axis. This has been reported after surgical and medical treatment of Cushing's disease, withdrawal of long-term steroid therapy, initial presentation of Addison’s disease, or relative glucocorticoids deficiency [11]. Cortisol excess increases CSF production and reduces its absorption, hence increasing intracranial pressure [12]. Another possible mechanism is the expression of both mineralocorticoid responsive epithelial sodium channel receptors on the basolateral membrane of the CSF producing epithelial cells of the choroid plexus as well as the expression of 11-beta hydroxysteroid dehydrogenase type 1 enzyme, which is a bidirectional enzyme that mainly functions to convert the inactive cortisone to active cortisol. These mechanisms play a role in maintaining the balance between CSF production and absorption [13,14].

In this case, the patient presented some clinical findings that are rarely associated with Cushing's disease, combined with a radiological feature that masked the true diagnosis. Our patient presented with significant weight loss, rather than central obesity, which is normally associated with Cushing’s disease. Although possible, the increase in ACTH due to Cushing's disease is not sufficient to cause hyperpigmentation, which is a classical finding of Addison's disease, where the entire adrenal cortex is usually affected due to an autoimmune destruction; however, the zona glomerulosa of the adrenal cortex produces aldosterone and its deficiency would lead to hyperkalemia [15]. Our patient presented with both hyperpigmentation and hypokalemia.

## Conclusions

EST syndrome is an uncommon radiological finding of apparently EST that has been reported in association with ICHTN. The latter has also been seen in association with Cushing's disease/syndrome. This is likely to result from glucocorticoid excess-induced change in CSF flow dynamics. EST has been infrequently described in association with Cushing's disease. This association has a clinical implication as it is likely to obscure the visualization of pituitary lesions responsible for Cushing's disease, contribute to diagnostic uncertainty, and increase the risk of healthy pituitary tissue injury and the risk of postoperative complications including CSF leakage.
